# Perfluorohexyloctane: More than Meets the Eye?

**DOI:** 10.1021/acs.chemrestox.5c00507

**Published:** 2026-02-02

**Authors:** Andi Alijagic, Matej Orešič, Tuulia Hyötyläinen

**Affiliations:** † Man-Technology-Environment (MTM) Research Centre, School of Science and Technology, 6233 Örebro University, SE-701 82 Örebro, Sweden; ‡ Inflammatory Response and Infection Susceptibility Centre (iRiSC), Örebro University, Örebro SE-701 82, Sweden; § School of Medical Sciences, Faculty of Medicine and Health, Örebro University, SE-701 82 Örebro, Sweden; ∥ Centre for Biotechnology, University of Turku and Åbo Akademi University, FI-20520 Turku, Finland; ⊥ Department of Life Technologies, University of Turku, FI-20014 Turku, Finland

## Abstract

Perfluorohexyloctane (F6H8) is a semifluorinated alkane increasingly used in medical applications. Emerging evidence, however, indicates that this compound can persist in biological systems and influence cellular processes. These observations suggest that the exceptional stability of F6H8, while beneficial for medical performance, may also have implications for long-term biological and health outcomes.

## Graphical abstract

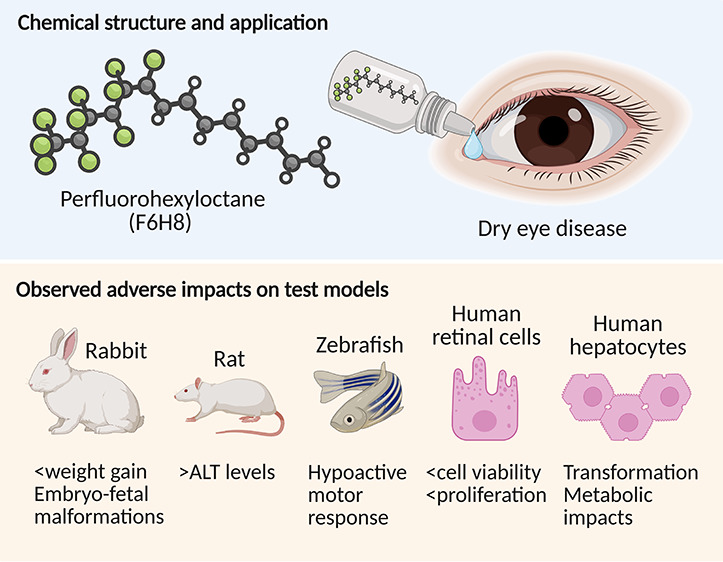

Perfluorohexyloctane (F6H8) is a semifluorinated alkane that has attracted attention for its expanding use in ophthalmology.^1^ Its unusual amphiphobic character, repelling both aqueous and lipid environments, enables it to spread uniformly over biological surfaces, stabilize tear films, and form transparent protective layers. These properties, coupled with its physicochemical stability and negligible volatility, have led to its approval as an ingredient in ophthalmic formulations for dry eye disease and as an intraocular tamponade in retinal procedures. Because of its low reactivity and general tolerance in preclinical and clinical (short-term topical administration) studies, F6H8 has been widely considered biocompatible.^2^ Yet, a closer look at the available and limited data suggests that its interaction with biological systems is more complex than the label of chemical stability might imply.
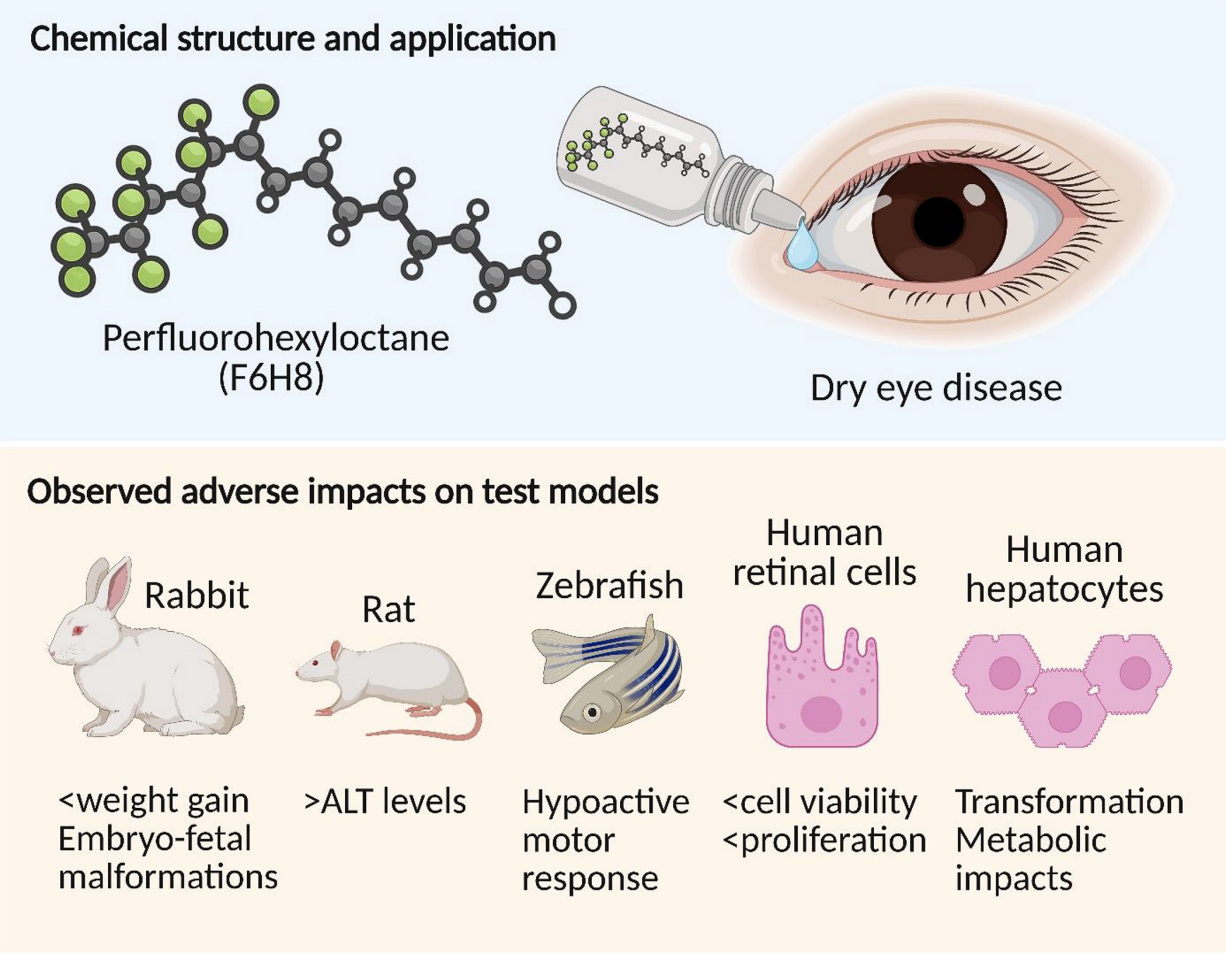


Rather than introducing new hazard end points, this ToxWatch article aims to integrate dispersed evidence from regulatory toxicology, experimental models, and pharmacokinetic studies to reassess the biological behavior of F6H8. By shifting the focus from acute toxicity to biological persistence, lipid-associated interactions, and emerging metabolic effects, we highlight an underexplored dimension of F6H8 safety that is not captured by existing reviews centered on clinical efficacy or short-term tolerability.

To date, information on the biological effects of F6H8 from *in vivo* studies remains limited, comprising mainly nonclinical pharmacology and toxicology evaluations in rabbits and rats.^3^ The end points investigated in these studies include general toxicity and embryo-fetal developmental (EFD) effects. In a 26-week ocular toxicity study in rabbits, repeated ocular instillation of an F6H8 ophthalmic solution at high daily doses was well tolerated and produced no ocular or systemic signs of toxicity.^3^ Similarly, a 28-day oral toxicity study in rats reported no treatment-related effects following daily administration of up to 2000 mg/kg. An EFD study in rats, conducted during the period of organ development, found no adverse effects on maternal health or fetal development at the same dosing levels.^3^ Altogether, these studies have supported the view that F6H8 poses little risk of acute toxicity under the studied exposure conditions.

Topical ocular administration represents the primary clinically relevant exposure route for F6H8, with limited expected systemic exposure. In contrast, oral and intravenous studies discussed here employ higher doses and are intended to probe systemic distribution, persistence, and biological interactions rather than to model clinical use, and should therefore be interpreted as tools for mechanistic insight and biological context rather than direct proxies for human exposure.

However, not all findings have been consistently reassuring. In an EFD study in rabbits, daily oral administration of F6H8 at doses between 250 and 1000 mg/kg/day led to abortions across all treatment groups and dose-related reductions in maternal weight gain and food consumption. Reduced fecal output and soft stool were observed, together with decreased fetal weights as compared to control animals. Although there was no evidence of increased embryo-fetal death or delayed skeletal ossification, a higher incidence of external, visceral, and skeletal malformations was reported in the treated groups.^3^ Additional studies have explored F6H8 exposure through alternative formulations and in different biological contexts. In a rat study using an F6H8-based propofol formulation, animals exhibited elevated alanine aminotransferase (ALT) levels without corresponding liver histopathological changes, suggesting subtle hepatic metabolic responses rather than overt toxicity.^4^ While this finding does not indicate overt hepatotoxicity and may reflect high-dose exposure conditions, it suggests a hepatic metabolic response that could be informative when interpreted alongside evidence of persistence or lipid-associated accumulation. *In vitro* investigations with human corneal endothelial and retinal pigment epithelial cells have shown that high concentrations of F6H8 reduce cell viability and proliferation;^5^ these effects likely reflect exposure levels exceeding typical clinical conditions but nevertheless indicate that F6H8 can influence cellular processes. These effects are believed to stem from the compound’s strong lipophilicity and its capacity to interact with lipid membranes, potentially disrupting membrane organization and intracellular lipid dynamics. In a zebrafish embryo model, F6H8 exposure was associated with a hypoactive photomotor response, indicating potential developmental neurotoxicity at early life stages.^6^ Taken individually, many of these findings are limited by high-dose or *in vitro* conditions; however, when considered collectively, they support the view that F6H8 engages biological systems in ways not fully captured by standard acute toxicity assessments.

Pharmacokinetic and tissue distribution studies further support the notion of biological persistence. In rabbits receiving ocular doses of radiolabeled F6H8, measurable plasma concentrations were detected within hours and declined slowly over 24 h, implying incomplete elimination and possible accumulation with repeated exposure.^7^ Investigations of structurally related semifluorinated alkanes, such as F6H10E, have demonstrated long organ retention times, with hepatic half-lives approaching several weeks and preferential distribution to lipid-rich tissues such as liver and spleen.^8^ Notably, analogous nonfluorinated alkanes are readily metabolized in the liver to carboxylic acids of the same chain length,^9^ underscoring how fluorination alters biological fate.

The emerging evidence suggests that while F6H8 does not exhibit acute toxicity, it can persist in biological systems and influence cellular processes through its strong affinity for lipid-rich environments. Recent findings indicate that F6H8 can alter several metabolic pathways, including amino acid metabolism, fatty acid turnover, phospholipid remodeling, and other processes linked to cellular energy balance, and may undergo limited biotransformation into fluorinated metabolites that retain highly stable perfluorinated moieties and physicochemical characteristics reminiscent of per- and polyfluoroalkyl substances (PFAS).^10^ Although the precise structures and *in vivo* relevance of these products have yet to be established, current data suggest that F6H8 may not be entirely metabolically inert. These observations (a) suggest that the biological fate of F6H8 is more complex than previously assumed and (b) broaden our understanding of how highly stable fluorinated molecules interact with living systems.

From a regulatory and environmental perspective, F6H8 represents a new generation of functional fluorochemicals that occupy a space between medical innovation and environmental concern. Although F6H8 is not classified as a PFAS, as it lacks a fully perfluorinated carbon backbone and does not meet current regulatory definitions, it shares several characteristics relevant to PFAS risk considerations. These include high chemical stability, low degradability, and preferential partitioning into lipid-rich environments, which together raise questions about persistence and potential bioaccumulation. Its use in medical contexts is subject to rigorous safety testing, yet these tests often focus on short-term tolerability and acute end points. The persistence and potential bioactivity observed in experimental models underscore the value of complementing such evaluations with studies that address distribution, retention, and cumulative effects. Notably, U.S. FDA approval documents state that F6H8 is not metabolized by human liver microsomes, but no supporting data or peer-reviewed evidence have been made publicly available, leaving important aspects of its metabolic fate unresolved.

F6H8 thus provides a timely example for the ongoing refinement of safety assessment frameworks for stable fluorinated compounds. Its application demonstrates the technological benefits of chemical stability, clarity, low volatility, and inertness in formulation, yet also highlights the biological implications of that same stability. Continued interdisciplinary research will be essential to clarify whether F6H8 and related molecules remain biologically quiescent over time or participate in more subtle, cumulative processes that challenge current definitions of safety. Ultimately, F6H8 reminds us that stability and safety are not synonymous. The absence of reactivity does not preclude interaction, and the persistence that enables medical utility may also sustain biological presence. As the use of fluorinated materials expands, recognizing these nuances will be crucial for designing compounds that are both effective and sustainable—chemicals that meet functional needs without leaving an enduring biological or environmental footprint. This integrative perspective complements the existing literature by emphasizing persistence-driven biological interactions as a critical consideration for the safety assessment of highly stable fluorinated compounds.
